# Supplementary material for: Reading the Score of the Air—Change in Airborne Microbial Load in Contrast to Particulate Matter during Music Making
Source: Int J Environ Res Public Health. 2022 Aug 12;19(16):9939. doi: 10.3390/ijerph19169939 (PMC9408517; doi:10.3390/ijerph19169939)
Supplement: Supplementary file 1 [file ijerph-19-09939-s001.zip › ijerph-1760606-supplementary.pdf]

International Journal of
Environmental Research and Public Health

MDPI

Supplement Material

Reading the score of the air - Change in Airborne Microbial Load in Contrast to Particulate Matter during Music Making

Birte Knobling [1], Gefion Franke [1], Lisa Beike[1], Timo Dickhuth[1], and Johannes K. Knobloch[1]*

[1] Institute for Medical Microbiology, Virology and Hygiene, University Medical Center Hamburg-Eppendorf, Hamburg, Germany
* Correspondence: j.knobloch@uke.de; Tel.: +49-40-741051720

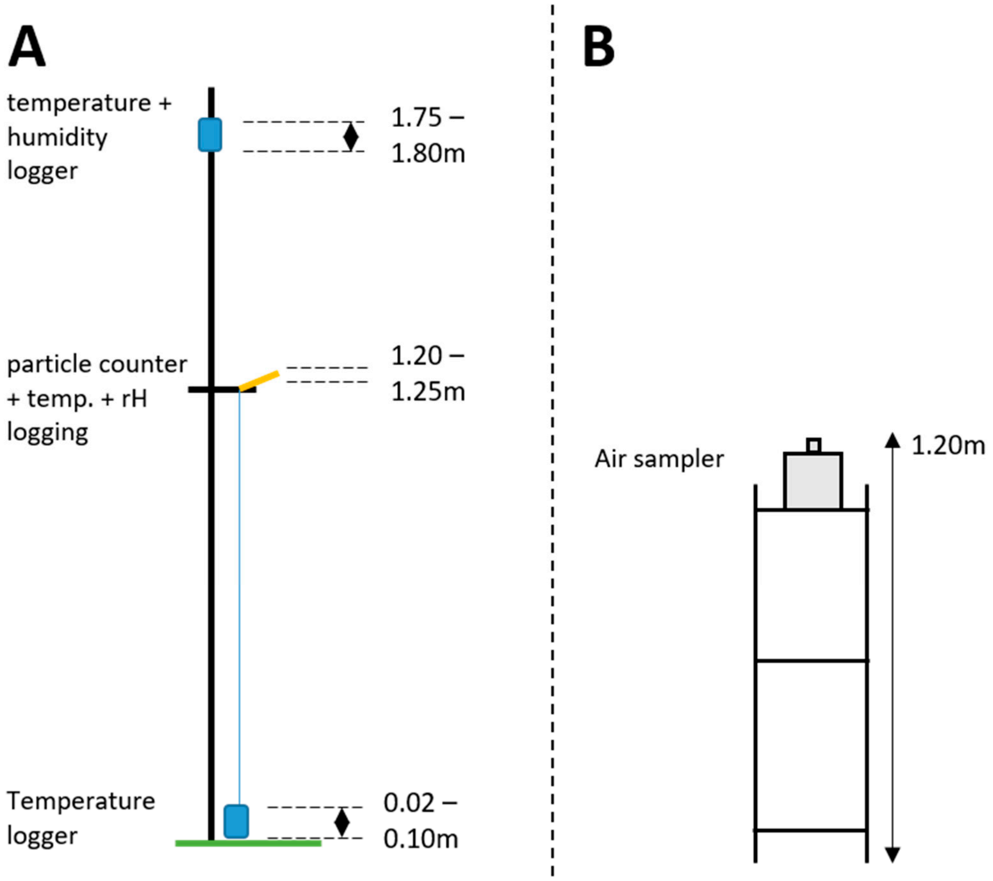

Figure S1: **Measurement setup particle counting (A) and air sampling (B).** Log-tag loggers were attached to each laboratory stand in the area of the floor (0,02 – 0.10 m) and above the head height of seated persons (1.75 – 1.80 m). The room temperature (temp.) was recorded at both positions and the relative humidity (rH) was also recorded at the head height position. In a height of 1.20-1.25 m the particle counter, also is logging temp. and rH, was positioned. The air sampler (B) was placed on a rack, so that the intake opening is at a height of approx. 1.20 m.

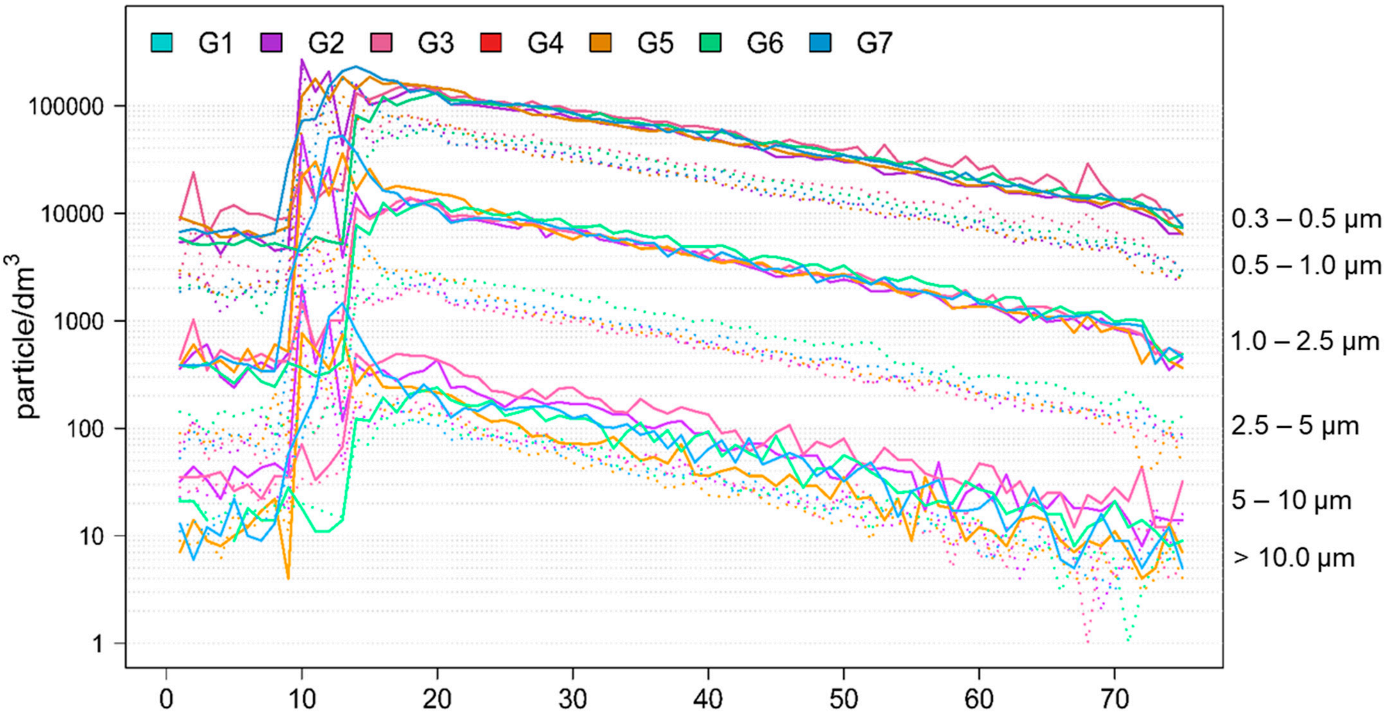

Figure S2: **Preliminary examination of particle counters.** The course of the particle load in the indoor air in a period of 80 minutes within an office room was determined using seven calibrated laser particle counter PC200 (Trotec, Heinsberg, Germany) with the analysis method "differential". The different colors displayed the seven devices (G1 – G7). Particle sizes in the range 0.3-0.5 µm, 1.0-2.5 µm, 5.0-10.0 µm are shown as solid lines and 0.5-1.0 µm, 2.5-5.0 µm, >10.0 µm as dashed lines. After approximately 10 minutes, aerosols were introduced into the room for about 5 minutes using aerosol generator ATM 228 (Topas GmbH, Dresden, Germany). Then, an air purification device (KA-520-L, Kampann, Lingen, Germany) were used to degrade the particle load of the indoor air. Meanwhile, each particle counter determines the size and amount of particles per minute contained in 1 L indoor air volume.

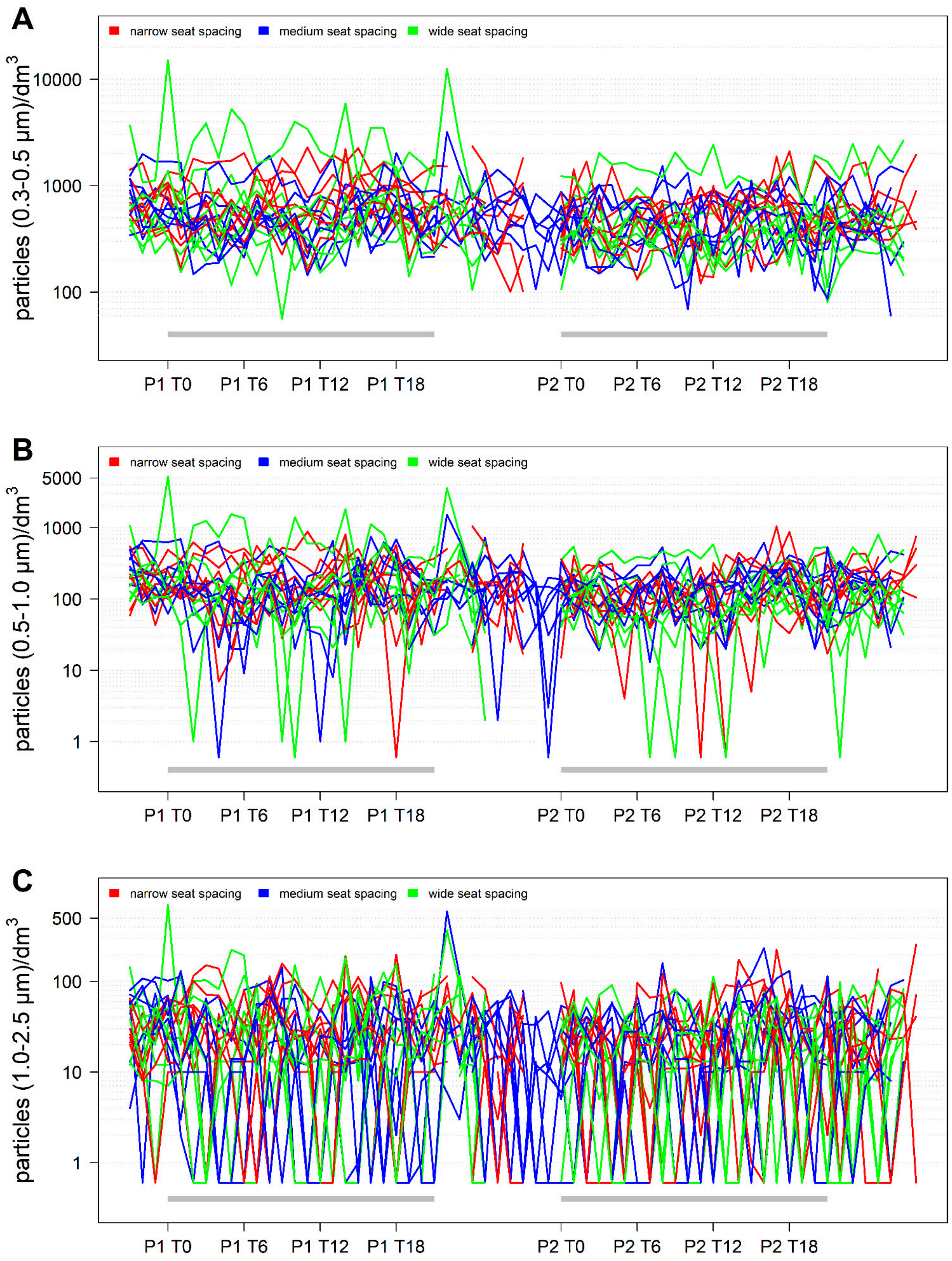

Figure S3. Particle loads during the presence of the orchestra. Shown are particle sizes of 0.3 µm (A), 0.5 µm (B), and 1.0 µm (C) at narrow (red), medium (blue), and wide (green) seating distances. The detection limit for all particle sizes is 1 particle/dm3. The labels of the ordinates indicate the time points of the germ count determination by means of air samplers within the measurement periods for the first and the second performance (P1 and P2) of the musical pieces. The broad gray lines mark the duration of the played music pieces. The localization of the measuring instruments was changed in each case between the P1 and P2. Detailed descriptions of the of the results at the individual measurement positions can be found in the Supplementary Figures S9-13

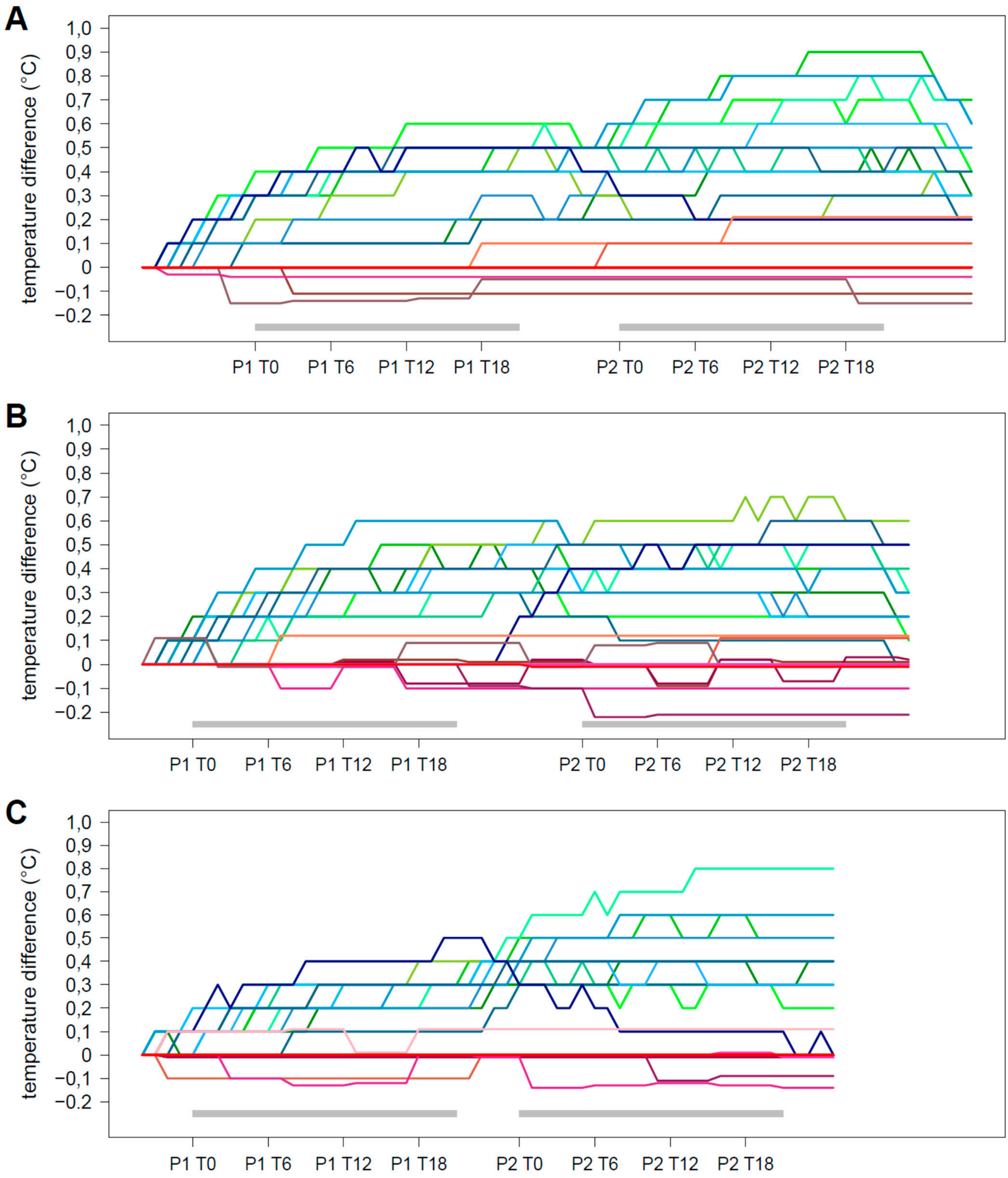

Figure S4. Temperature curves at the measuring points. The temperature curves at the measurement points near the floor (green shades), the measurement points in the zone above the seated orchestra members (blue shades) and the measurement points of the building control system of the concert hall (red shades) are displayed. The labels of the partial figures correspond to the three measurement phases with narrow (A), middle (B) and wide (C) seating arrangement. The labels of the ordinates indicate respectively the times of the germ count determination by means of air samplers within the measurement periods for the first and the second play (P1 and P2) of the musical pieces. The broad gray lines mark the duration of the played music pieces. Due to the accuracy of the measuring instruments used, only an orienting measurement was possible. However, there was no temperature increase of more than 1 °C during any of the orchestra's stays on stage (see also Figure S6).

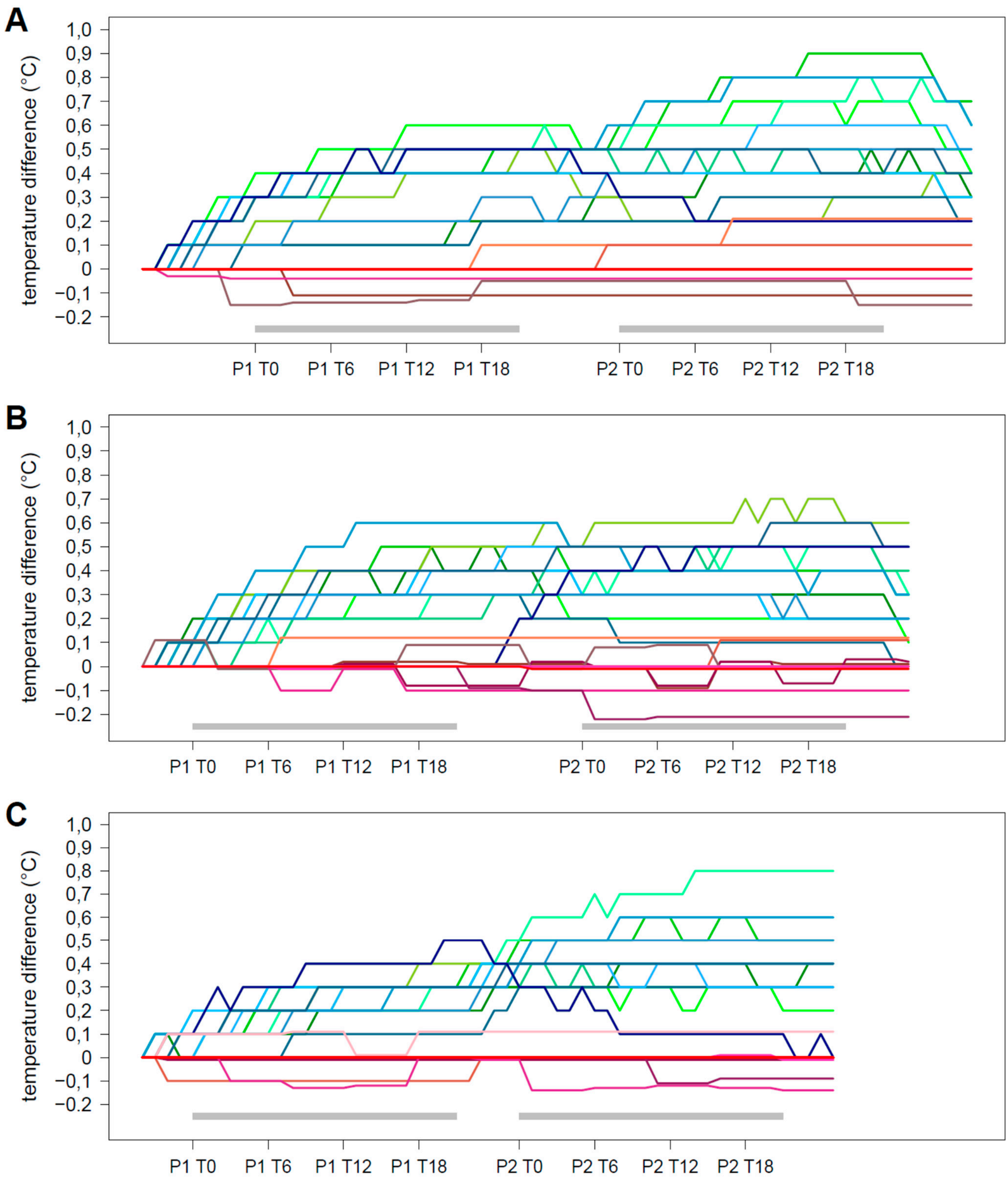

Figure S5. Temperature differences of time at the measuring points. The temperature differences over the time of presence of the orchestra at the stage for the measurement points near the floor (green shades), the measurement points in the zone above the seated orchestra members (blue shades) and the measurement points of the building control system of the concert hall (red shades) are displayed. The labels of the partial figures correspond to the three measurement phases with narrow (A), middle (B) and wide (C) seating arrangement. The labels of the ordinates indicate respectively the times of the germ count determination by means of air samplers within the measurement periods for the first and the second play (P1 and P2) of the musical pieces. The broad gray lines mark the duration of the played music pieces. Due to the accuracy of the measuring instruments used, only an orienting measurement was possible. However, there was no temperature increase of more than 1 °C during any of the orchestra's stays on stage.

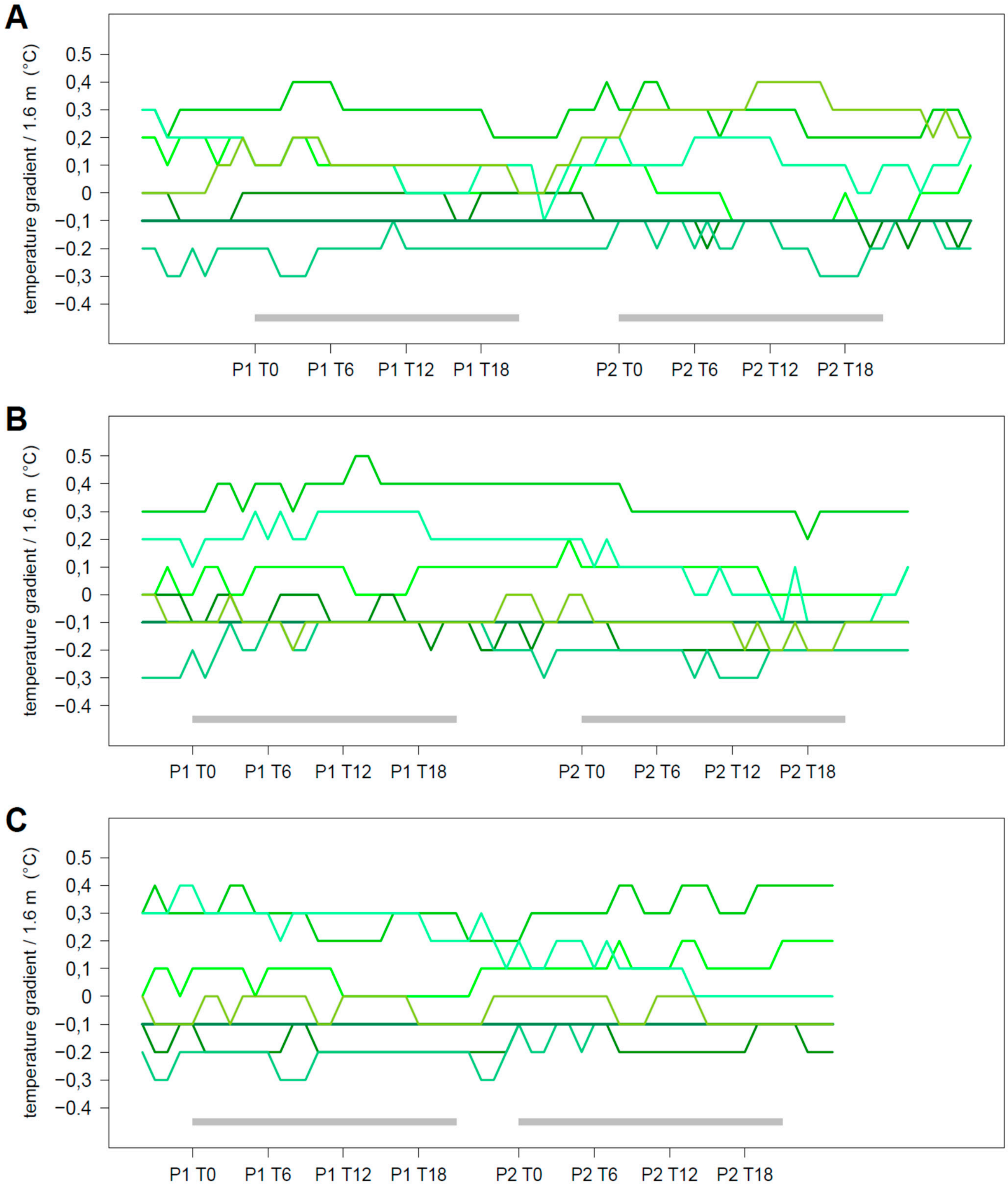

Figure S6. Temperature gradient at the measuring points. The temperature gradient between the measurement points near the floor and the measurement points in the zone above the seated orchestra members are displayed for the paired instruments respective. The labels of the partial figures correspond to the three measurement phases with narrow (A), middle (B) and wide (C) seating arrangement. The labels of the ordinates indicate respectively the times of the germ count determination by means of air samplers within the measurement periods for the first and the second play (P1 and P2) of the musical pieces. The broad gray lines mark the duration of the played music pieces. Due to the accuracy of the measuring instruments used, only an orienting measurement was possible. A temperature gradient critical for displacement ventilation was not evident at any time, as no temperature difference of more than 1 °C could not be observed with the temperature sensors on the stage with a height difference of approx. 1.6 m.

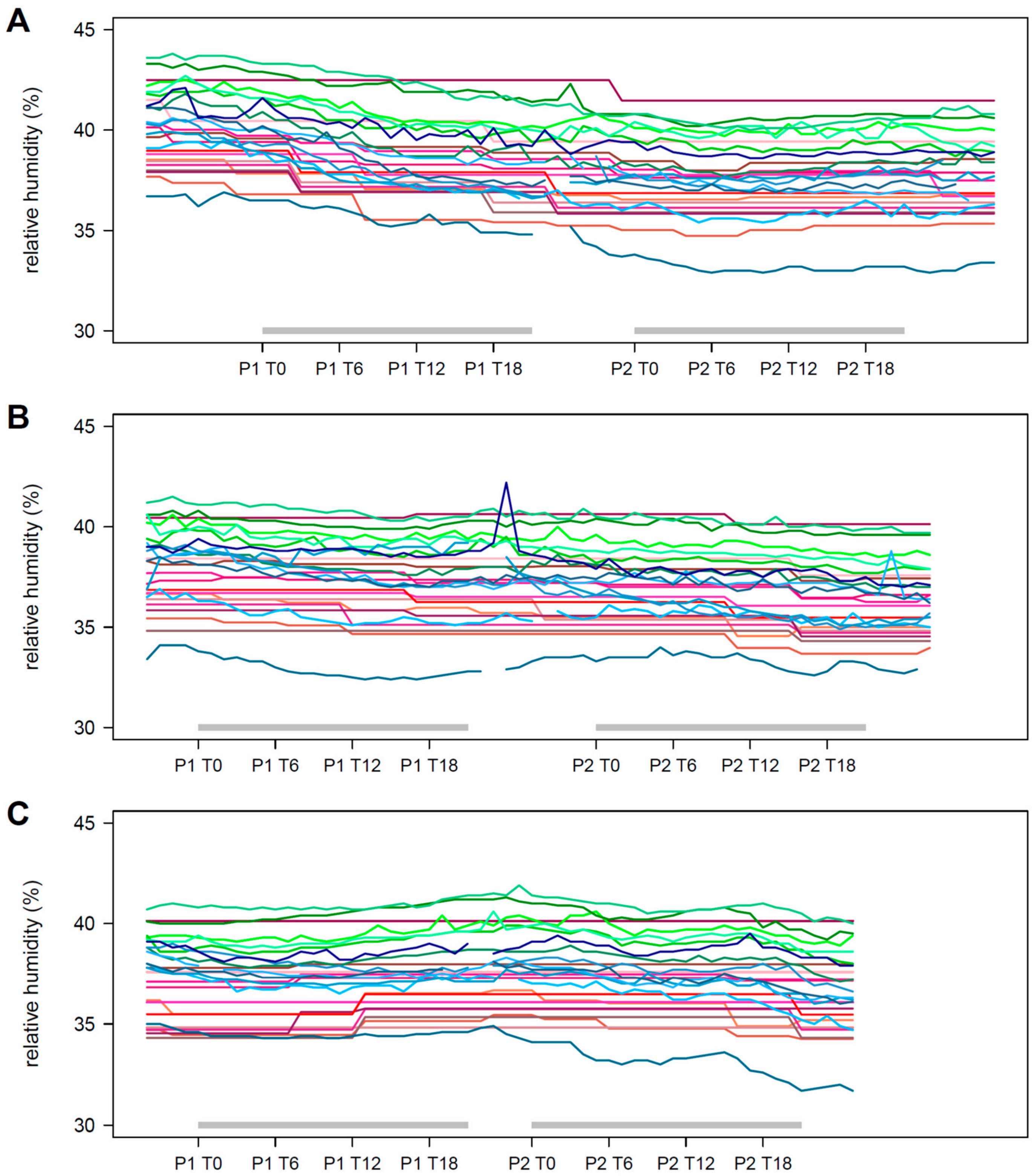

Figure S7. Relative humidity at the measuring points. The relative humidity at the measurement points near the floor (green shades), the measurement points in the zone above the seated orchestra members (blue shades) and the measurement points of the building control system of the concert hall (red shades) are displayed. The labels of the partial figures correspond to the three measurement phases with narrow (**A**), middle (**B**) and wide (**C**) seating arrangement. The labels of the ordinates indicate respectively the times of the germ count determination by means of air samplers within the measurement periods for the first and the second play (P1 and P2) of the musical pieces. The broad gray lines mark the duration of the played music pieces.

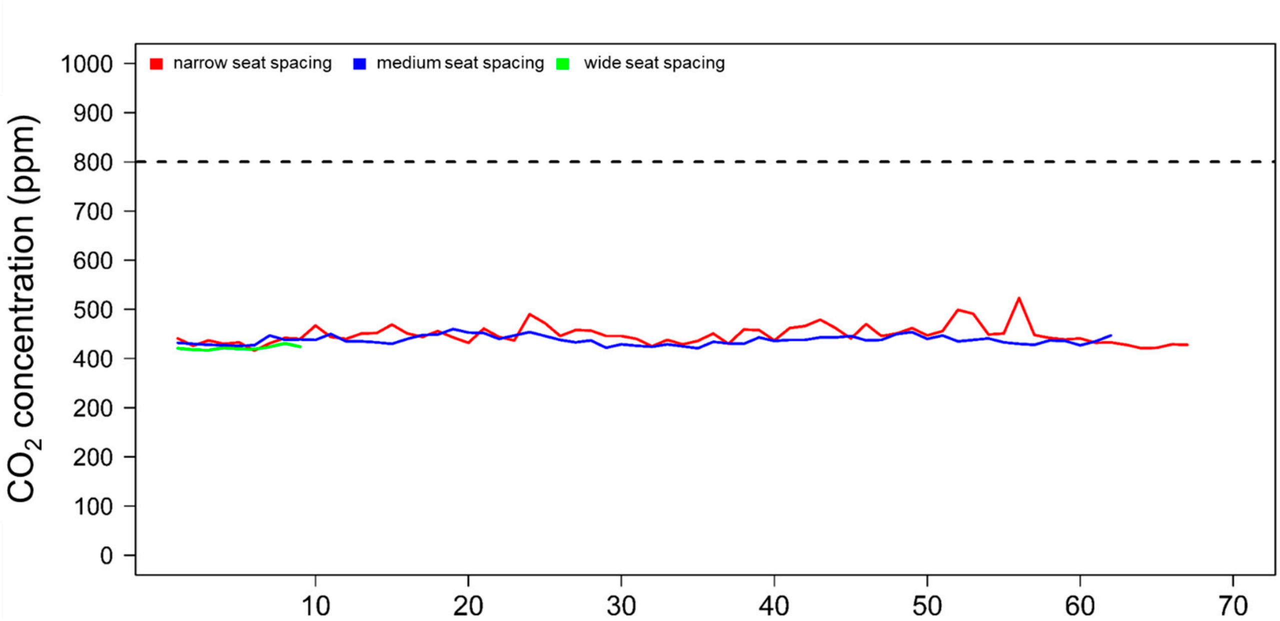

Figure S8. Content of CO_2 concentration (ppm) in indoor air. Continuous CO_2 measurement was performed at one position in the middle of the stage during presence of musician on stage. The labels of the x-ordinate showed the time in minutes. The different colors display various seating setups on stage. For technical reasons, only CO_2 values for the first 20 minutes are available for the wide setup. The dashed line displayed the recommended CO_2 value for good and safe indoor air quality

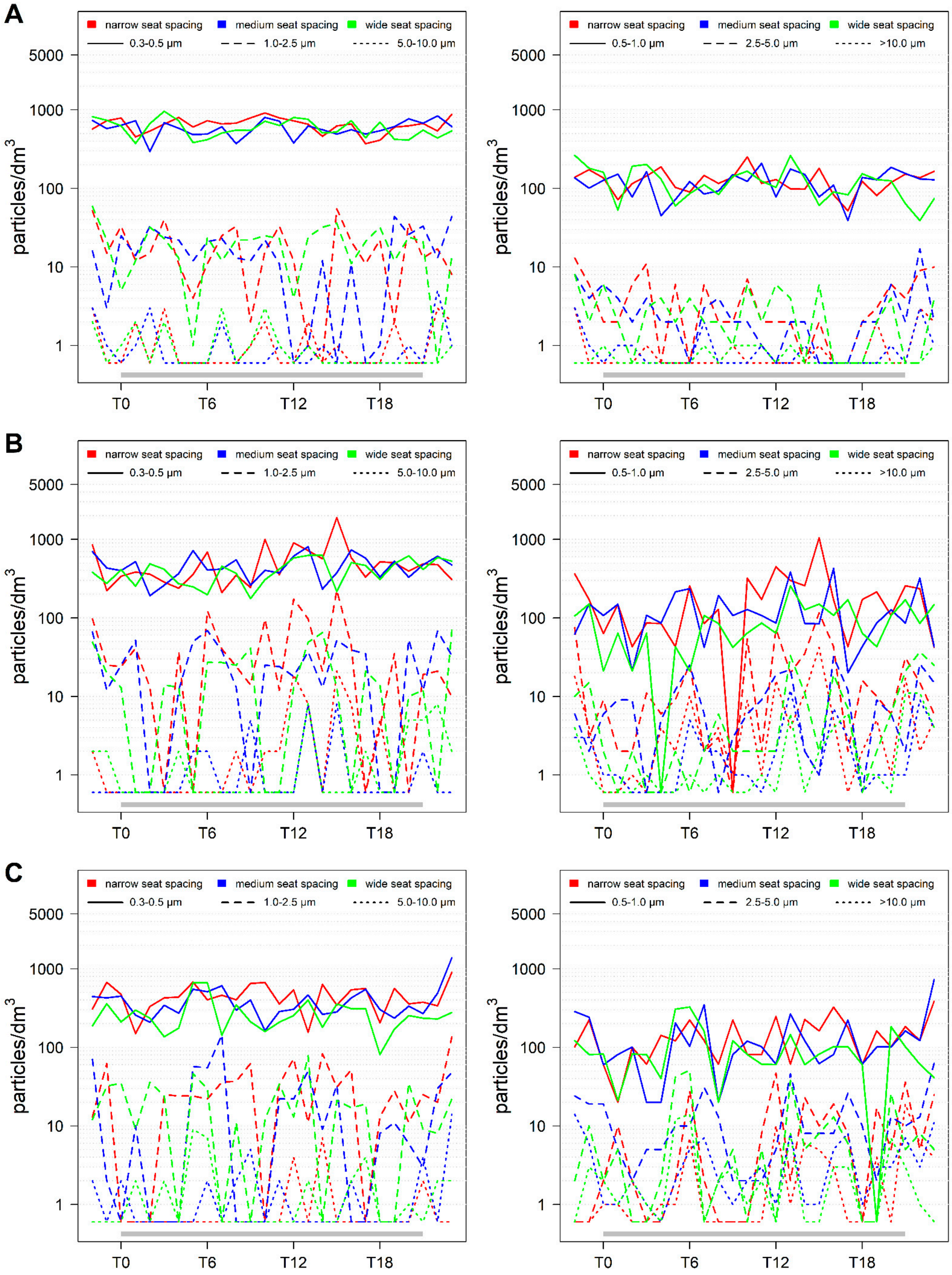

Figure S9. Results of particle measurement in zone 1. Particle sizes of 0.3 µm, 1.0 µm and 5.0 µm (left) and 0.5 µm, 2.5 µm and 10 µm (right) are displayed. The designations of the partial figures (**A** to **C**) correspond to the marking of the particle samplers in zone 1 of figure 1. The detection limit for all particle sizes is 1 particle/dm^3. The labels of the ordinates indicate the time of the start of the measurement of air sampling within the piece of music. The broad gray lines mark the duration of the played music pieces.

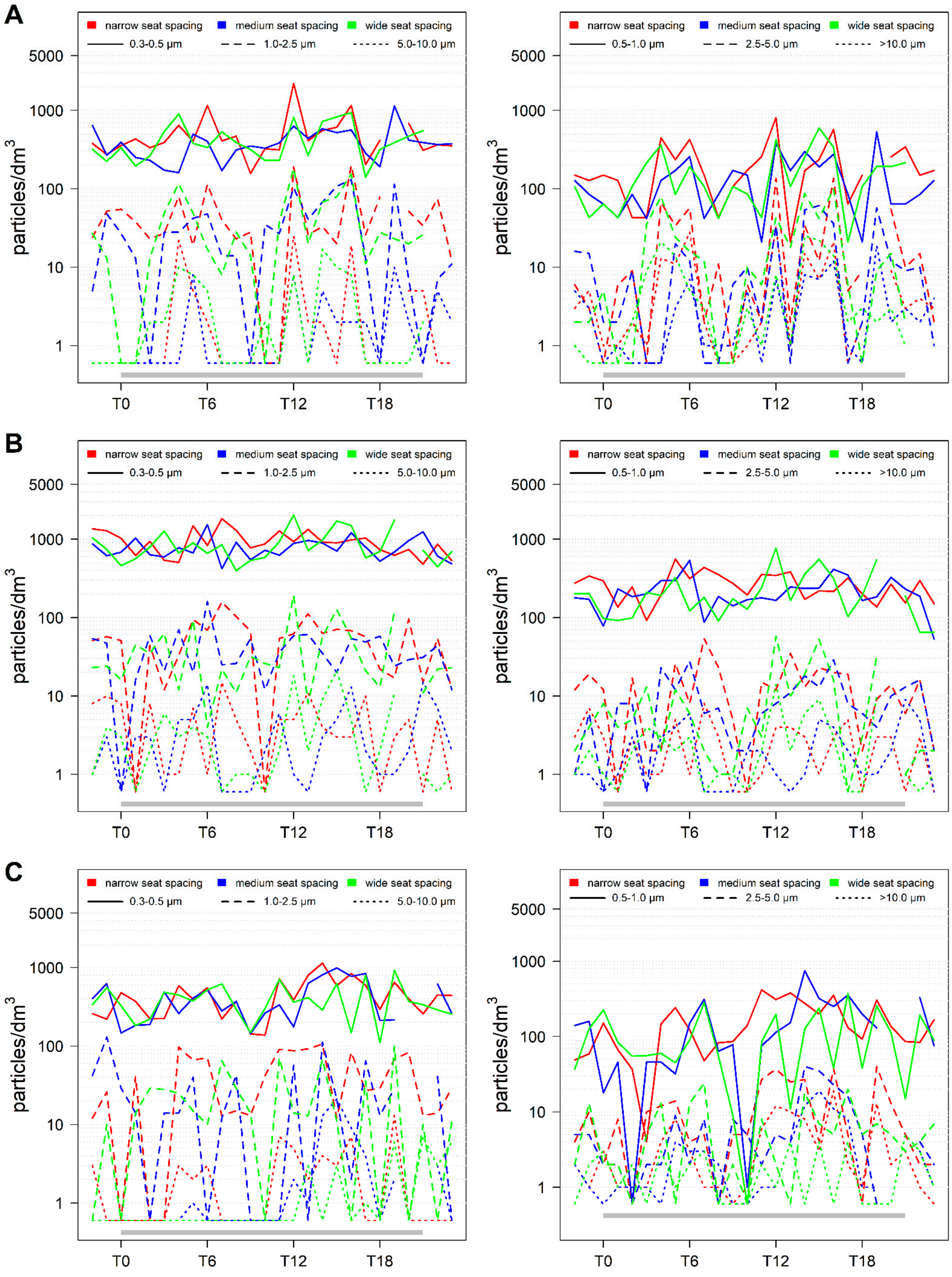

Figure S10. Results of particle measurement in zone 2. Particle sizes of 0.3 µm, 1.0 µm and 5.0 µm (left) and 0.5 µm, 2.5 µm and 10 µm (right) are displayed. The designations of the partial figures (**A** to **C**) correspond to the marking of the particle samplers in zone 2 of figure 1. The detection limit for all particle sizes is 1 particle/dm³. The labels of the ordinates indicate the time of the start of the measurement of air sampling within the piece of music. The broad gray lines mark the duration of the played music pieces.

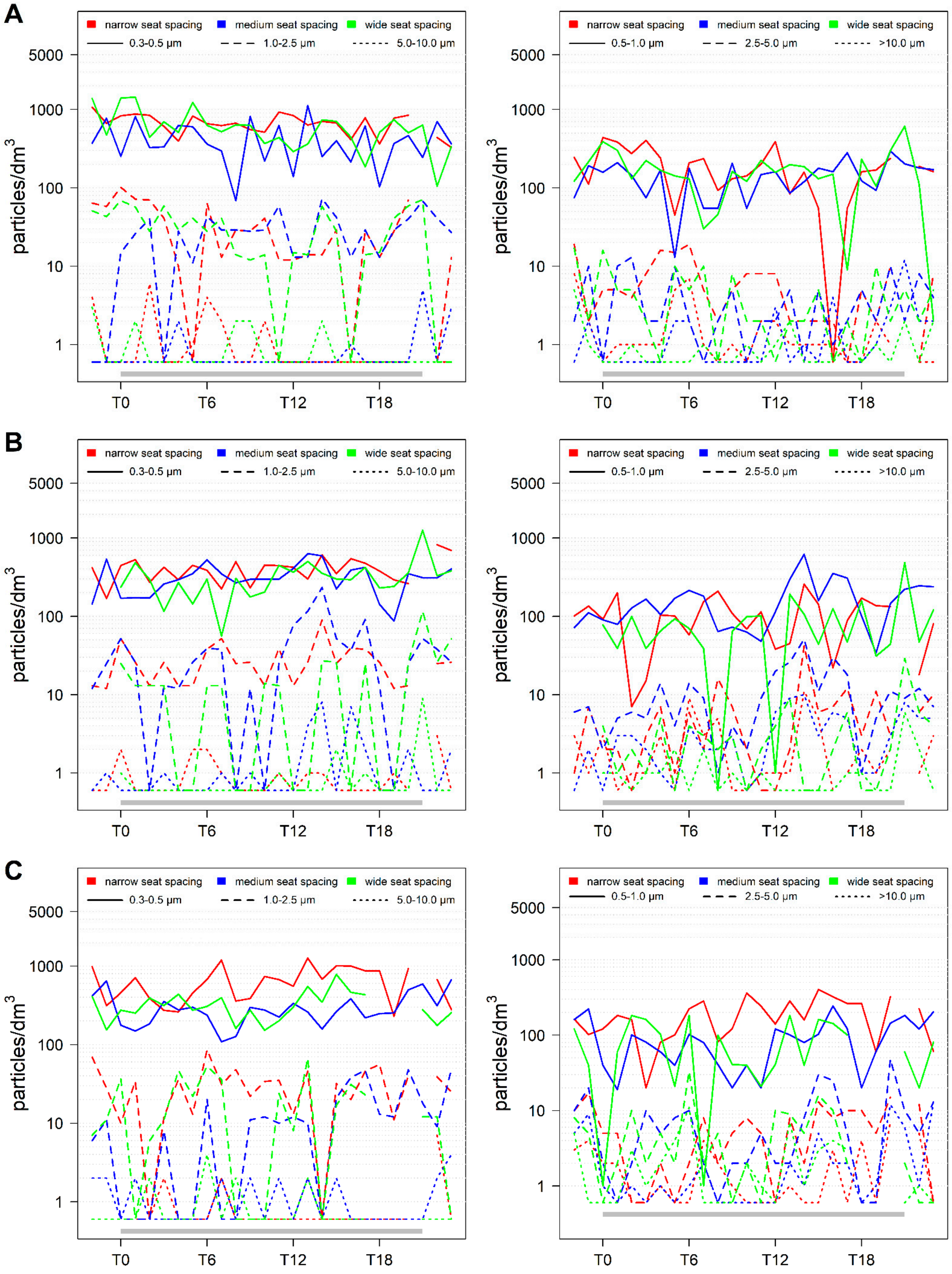

Figure S11. Results of particle measurement in zone 3. Particle sizes of 0.3 µm, 1.0 µm and 5.0 µm (left) and 0.5 µm, 2.5 µm and 10 µm (right) are displayed. The designations of the partial figures (A to C) correspond to the marking of the particle samplers in Zone 3 of Figure 1. The detection limit for all particle sizes is 1 particle/dm3. The labels of the ordinates indicate respectively the times of the germ count determination by means of air samplers within the measurement periods during playing the musical piece. The broad gray lines mark the duration of the played music pieces.

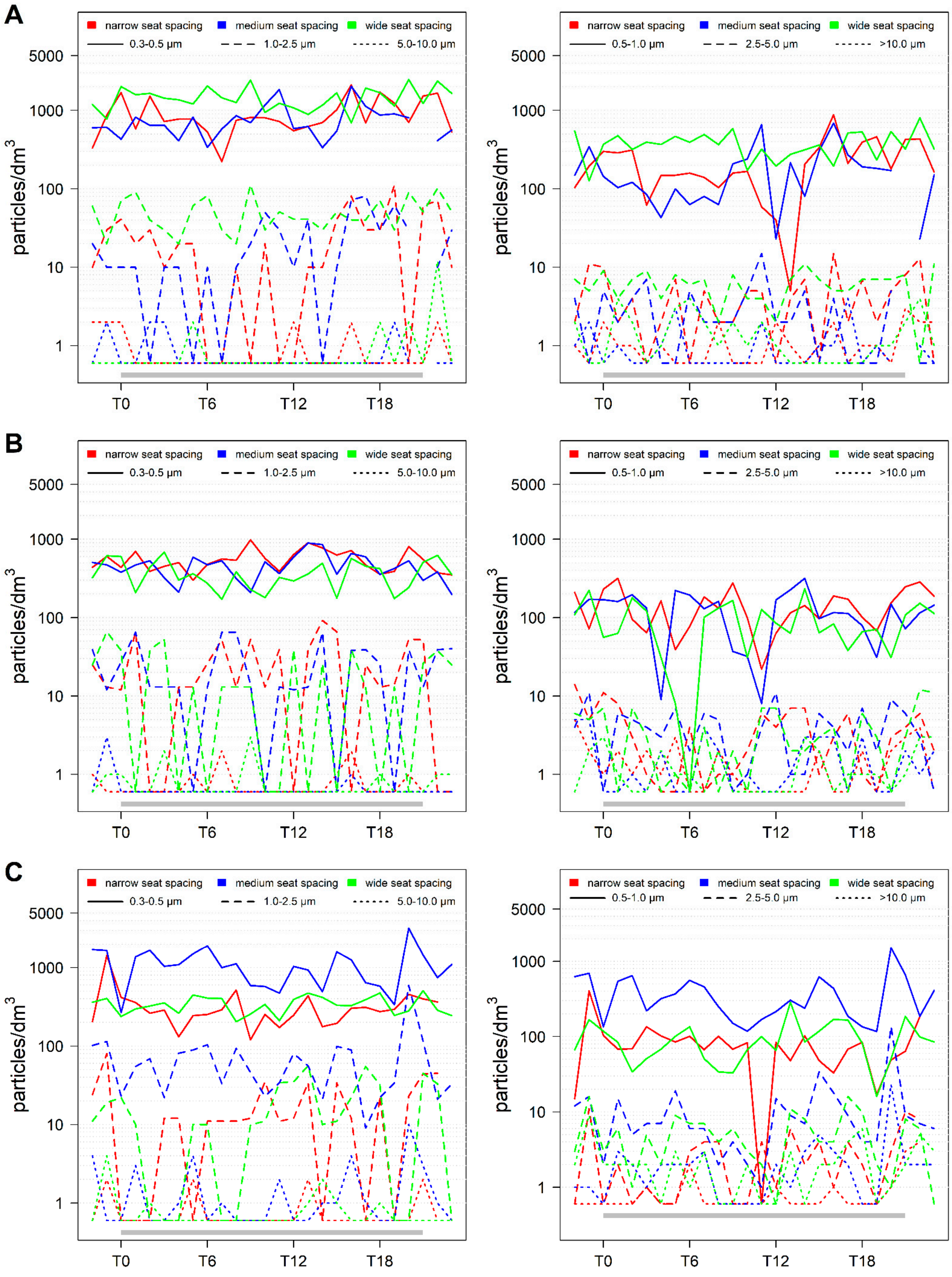

Figure S12. Results of particle measurement in zone 4. Particle sizes of 0.3 µm, 1.0 µm and 5.0 µm (left) and 0.5 µm, 2.5 µm and 10 µm (right) are displayed. The designations of the partial figures (**A** to **C**) correspond to the marking of the particle samplers in zone 4 of figure 1. The detection limit for all particle sizes is 1 particle/dm³. The labels of the ordinates indicate the time of the start of the measurement of air sampling within the piece of music. The broad gray lines mark the duration of the played music pieces.

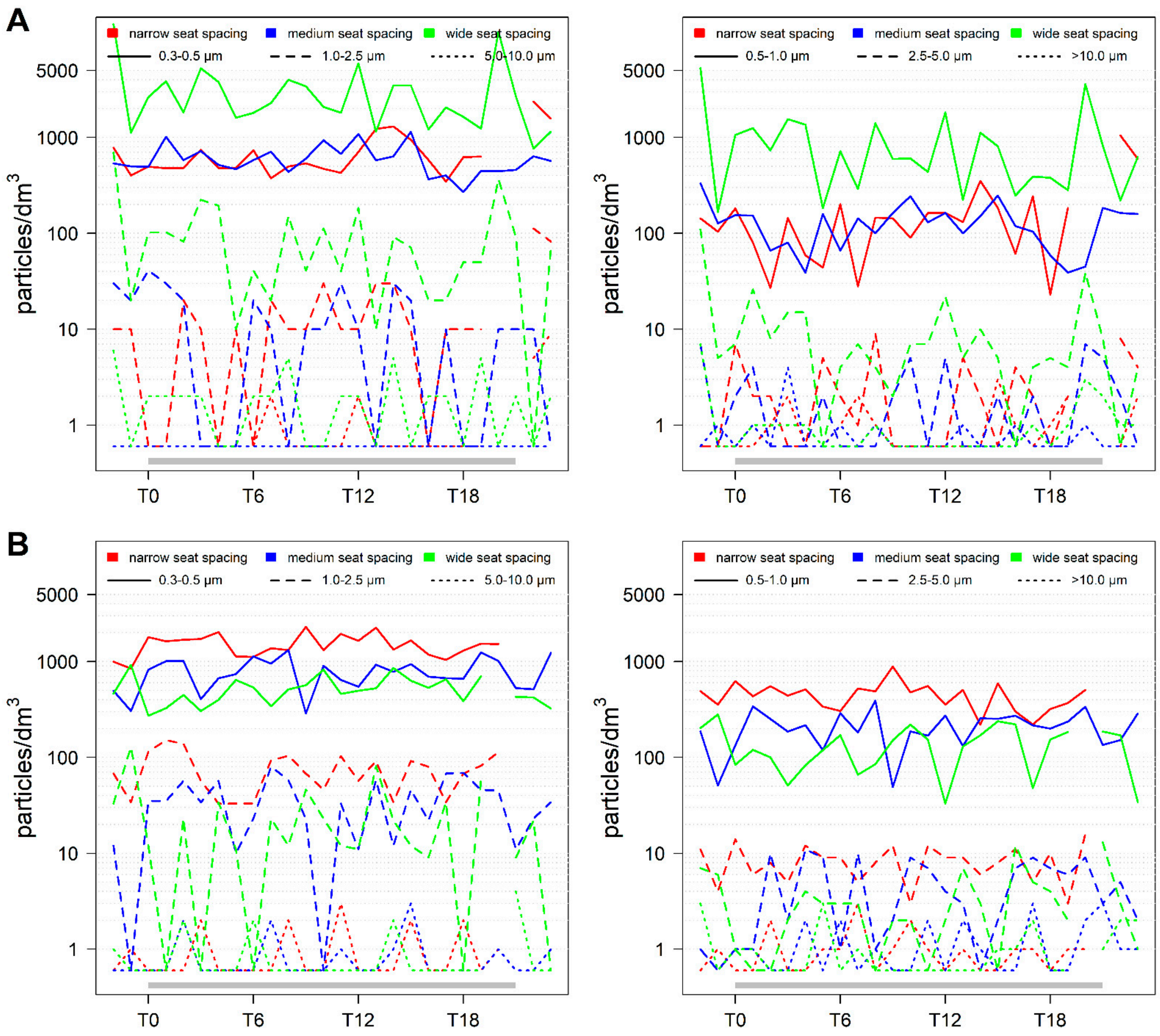

Figure S13. Results of particle measurement in zone 5. Particle sizes of 0.3 µm, 1.0 µm and 5.0 µm (left) and 0.5 µm, 2.5 µm and 10 µm (right) are displayed. The designations of the partial figures (**A** to **C**) correspond to the marking of the particle samplers in zone 5 of figure 1. The detection limit for all particle sizes is 1 particle/dm³ The labels of the ordinates indicate the time of the start of the measurement of air sampling within the piece of music. The broad gray lines mark the duration of the played music pieces.

Table S1. Results of statistical analysis. The results and adjusted p-values of pairwise Bonferroni corrected Welch-t-tests are displayed.

<table>
<tr><td>Size</td><td colspan="3">Particle size 0.3 µm</td><td colspan="3">Particle size 0.5 µm</td><td colspan="3">Particle size 1.0 µm</td></tr>
<tr><td>distances</td><td>Narrow</td><td>medium</td><td>Wide</td><td>narrow</td><td>medium</td><td>wide</td><td>narrow</td><td>medium</td><td>wide</td></tr>
<tr><td colspan="10">H_0: group1: particle load empty stage is equal to group 2: particle load narrow, medium, wide;
H_1: group1: particle load empty stage is different to group 2: particle load narrow, medium, wide;</td></tr>
<tr><td>result</td><td>ns</td><td>ns</td><td>ns</td><td>ns</td><td>ns</td><td>ns</td><td>ns</td><td>ns</td><td>ns</td></tr>
<tr><td>p.adj</td><td>0.129</td><td>0.109</td><td>0.087</td><td>0.284</td><td>0.273</td><td>0.07</td><td>0.081</td><td>0.077</td><td>0.804</td></tr>
<tr><td colspan="10">H_0: group1: control position on stage, direction approx. to audience area is equal to group 2: particle load during playing
H_1: group1: control position on stage, direction approx. to audience area is different to group 2: particle load during playing</td></tr>
<tr><td>result</td><td>ns</td><td>ns</td><td>***</td><td>**</td><td>****</td><td>ns</td><td>*</td><td>****</td><td>ns</td></tr>
<tr><td>p.adj</td><td>1</td><td>1</td><td>0.0004</td><td>0.004</td><td>2e-6</td><td>1</td><td>0.003</td><td>2.4e-7</td><td>0.35</td></tr>
<tr><td colspan="10">H_0: group1: particle load narrow distances is equal to group 2: particle load medium distances
H_1: group1: particle load narrow distances is different to group 2: particle load medium distances</td></tr>
<tr><td>result</td><td colspan="3">**</td><td colspan="3">ns</td><td colspan="3">ns</td></tr>
<tr><td>p.adj</td><td colspan="3">0.009</td><td colspan="3">1</td><td colspan="3">1</td></tr>
<tr><td colspan="10">H_0: group1: particle load narrow distances is equal to group 2: particle wide distances
H_1: group1: particle load narrow distances is different to group 2: particle wide narrow distances</td></tr>
<tr><td>result</td><td colspan="3">****</td><td colspan="3">****</td><td colspan="3">**</td></tr>
<tr><td>p.adj</td><td colspan="3">1.7e-12</td><td colspan="3">6.2e-8</td><td colspan="3">0.002</td></tr>
<tr><td colspan="10">H_0: group1: particle load medium distances is equal to group 2: particle load wide distances
H_1: group1: particle load medium distances is different to group 2: particle load wide distances</td></tr>
<tr><td>result</td><td colspan="3">****</td><td colspan="3">****</td><td colspan="3">**</td></tr>
<tr><td>p.adj</td><td colspan="3">1.3e-5</td><td colspan="3">1.7e-7</td><td colspan="3">0.003</td></tr>
</table>
